# Detecting suspicious activities at sea based on anomalies in Automatic Identification Systems transmissions

**DOI:** 10.1371/journal.pone.0201640

**Published:** 2018-08-09

**Authors:** Jessica H. Ford, David Peel, David Kroodsma, Britta Denise Hardesty, Uwe Rosebrock, Chris Wilcox

**Affiliations:** 1 CSIRO Data61, Castray Esplanade, Hobart, Tasmania, Australia; 2 Skytruth, Shepherdstown, West Virginia, United States of America; 3 CSIRO Oceans and Atmosphere, Castray Esplanade, Hobart, Tasmania, Australia; University of Minnesota, UNITED STATES

## Abstract

Automatic Identification Systems (AIS) are a standard feature of ocean-going vessels, designed to allow vessels to notify each other of their position and route, to reduce collisions. Increasingly, the system is being used to monitor vessels remotely, particularly with the advent of satellite receivers. One fundamental problem with AIS transmission is the issue of gaps in transmissions. Gaps occur for three basic reasons: 1) saturation of the system in locations with high vessel density; 2) poor quality transmissions due to equipment on the vessel or receiver; and 3) intentional disabling of AIS transmitters. Resolving which of these mechanisms is responsible for generating gaps in transmissions from a given vessel is a critical task in using AIS to remotely monitor vessels. Moreover, separating saturation and equipment issues from intentional disabling is a key issue, as intentional disabling is a useful risk factor in predicting illicit behaviors such as illegal fishing. We describe a spatial statistical model developed to identify gaps in AIS transmission, which allows calculation of the probability that a given gap is due to intentional disabling. The model we developed successfully identifies high risk gaps in the test case example in the Arafura Sea. Simulations support that the model is sensitive to frequent gaps as short as one hour. Results in this case study area indicate expected high risk vessels were ranked highly for risk of intentional disabling of AIS transmitters. We discuss our findings in the context of improving enforcement opportunities to reduce illicit activities at sea.

## Introduction

The Automatic Identification System (AIS) is an automated vessel tracking and monitoring system used to identify and locate vessels. It operates via the electronic exchange of data with nearby vessels, ground-based AIS systems and satellites. It was purpose-developed initially as a collision avoidance tool, but is now increasingly used to monitor vessel activities both at sea and in port, with numerous other applications. Some of these applications include fishing vessel fleet monitoring and control [1], managing ship traffic, particularly in and around ports [2,1], safety (including search and rescue), environmental impacts from marine activities [3], fleet and cargo tracking [4], and maritime security [1,5,6].

While historically vessel coverage was mainly from land-based receivers, with the increase in satellite capability since the early 2000’s, vessels are increasingly monitored from satellites [2]. This has significantly increased the global coverage of AIS, which is mandatory for all vessels with a gross tonnage of 300 or more, as well as all commercial passenger vessels, regardless of size (based on IMO’s International Convention for the Safety of Life at Sea). The global coverage implied by these policies is a key feature. While alternatives such as satellite radar are important, their cost is prohibitive for global scale monitoring. In some contexts vessel monitoring systems have been specifically designed, such as fisheries vessel monitoring systems (VMS). However AIS still presents the only large scale actively transmitted data source, and is comparatively inexpensive with respect to other options.

However, there are a number of issues with using AIS for monitoring vessels. Vessel operators have control over the system, including the data that is transmitted, whether transmissions occur, at what interval, and if the transmissions are an accurate representation of the vessel’s position, bearing and speed. One notable challenge for AIS data is gaps in transmissions. While transmissions should occur at regular intervals (varying with speed), there are often substantial gaps in transmission. These occur for three basic reasons: 1) saturation of the system in locations with high vessel density; 2) less frequent transmissions due to irregular transmission or poor quality transmissions due to poor quality or faulty equipment on the vessel or receiver; and 3) longer gaps in transmission due to intentional disabling of AIS transmissions. To date, there has been considerable work on anomaly detection for vessel movements (for example see [7,8,9], however many of these approaches focus on AIS derived from terrestrial base stations. Extension of these methods to incorporate satellite AIS data and multiple vessels simultaneously would be complex. The approach developed here has a much broader focus across multiple vessels in the open ocean, for which a large part of the data is satellite AIS. In comparison to terrestrial AIS, the non-intentional dropouts are on the whole driven by spatial and temporal satellite coverage and signal collision related to the number of vessels in the statellite’s footprint at a given time. In such a context, non-intentional gaps can range from a few minutes to many hours and has a strong spatial and temporal component. In comparison, terrestrial AIS base stations are static, with less temporal variation and the non-intentional gaps are in the order of minutes.

We present an approach to addressing this AIS transmission gaps problem, using a spatial statistical model to describe typical transmission rates by vessels using counts of transmissions per day. This model can then be used to examine the time-series data of individual vessels to establish if a vessel’s transmission rate deviates from what is expected. We were particularly interested in developing criteria to distinguish between intentional disabling and poor quality equipment/bad installation or satellite receiver saturation because the former is a direct action taken by vessel operators and indicates they may be avoiding observation.

We developed a Generalized Additive Model (GAM) to model space-time variations in AIS gaps in transmissions, enabling discrimination between intentional disabling and other issues. We established a metric to provide a probabilistic rank of vessels given AIS transmission patterns. We then applied this system to a case-study in a region bounded by Australia, Indonesia and Papua New Guinea (PNG). We evaluate the capacity of the model to identify of areas with known AIS shut-offs and vessels previously identified as engaging in illegal activities. Finally, we discuss the application of our model in the context real-time identification of vessels avoiding observation.

## Methods

### Generalized Additive Models

Generalized Additive Models (GAM) [10] are widely used for modelling complex processes where there is no clear functional form, including application to spatial modeling. We used GAMs to capture both the temporal and spatial patterns of vessel transmission frequency and satellite/terrestrial receiver coverage to establish expected transmission rates in the study region. These statistical models are able to represent relationships between a response variable, e.g. number of polls per day, and a predictor variable, e.g. location, time, flag state, etc., using a mix of parametric terms as in traditional regression and nonparametric terms made of a combination of smooth functions [10]. These models are particularly useful for representing spatial patterns, as they allow fitting of a smoothly varying spatial component without having to specify its specific form–in essence, the statistical version of a map–in a context where other parametric and non-parametric terms can also be included. We used the *gam* function in the *mgcv* package [11] in the R statistical language [12].

A binomial GAM was used to explore the temporal and spatial probability of a successful AIS transmission in a one hour window, given location and day for one month. The binomial model corresponds to the number of successfully received AIS polls per day from a vessel. The GAM model formula used was:

Pr{r,f}=B(N,r,p)

$$\frac{log(p)}{log(p+1)}=s(longitude,latitude)+f(Day)$$ (1)

where the probability of r received and f failed transmissions is Binomial, with N total possible transmissions per day (here 24), and p probability of a transmission in a 60 minute interval. The probability of a transmission is the inverse logit transform of a smooth function with interaction between terms, s(.), incorporating the latitude and longitude of the vessel on average for the day and f(.) indicates factor term for day. Due to location of the study area, and associated coastal boundaries, a soap film smooth was used which allows the smooth function to have a boundary which is also fitted using the same predictor variables in the process [13]. The final model of latitude, longitude and day was used to model the spatial patterns, other relevant covariates could be included in future versions.

### Anomalous gaps

The transmission data of each one hour interval is considered as an event. The outcome of each event can be the receipt of a signal or a failure to receive a signal within the interval. For each one hour interval the probability of a vessel successfully transmitting given the day and its location can be predicted from the GAM. We can then calculate the overall probability of an exhibited sequence of gap/received transmissions across the month for each vessel. Missed events (i.e. all one hour events with no AIS data received) were assigned the location of the last AIS position.

It is important to note that we use two time scales for data analysis: the model uses data summarised to each day or 24 hour block (based on one hour intervals); whereas prediction of probability of gaps returns to the one hour interval level. Using this method, a gap is defined as any series (1 or more) of missed hourly intervals. This approach assumes that the average transmission probability for a day is representative of hourly intervals within that day.

Our GAM model captures the expected frequency of transmissions at a time in a given location. This expected frequency will reflect variation due to terrestrial receiver availability, satellite coverage, traffic density, and typical transmission for vessels operating in the area. We assume that intentional disabling of AIS transmitters will be signified by unusually long runs of non-transmission events that are unlikely given the normal behaviour of vessels in the location (e.g. multiple ‘missing events’ in a continuous fashion). Based on this assumption we calculate the probability of each run length L of non-transmission events for each vessel, using the probability of a successful transmission at that location and time, $\hat{p}$, from the fitted model for transmission probability (Eq 2). We call this probability the Probability of Gap (PoG):

$$PoG={p(1-p)}^{L}$$

$$StdPoG=\frac{{PoG}_{i}}{\sum_{i}{PoG}_{i}/N}$$ (2)

These probabilities are then combined over the available record for the vessel, by standardizing the probability of each gap by the average probability of gaps over the total record for the vessel (StdPoG, Eq 2). This standardization allows us to take account of the variation in the operation of transmitters across vessels. Thus a vessel with a poorly functioning AIS system may have a run of non-transmissions of improbable length, but if these improbable non-transmission events occur frequently, standardizing will suggest that long gaps are typical for the vessel.

### Model validation

To test how effective the model is at detecting abnormal gaps, we randomly selected one third of all vessels from the data and added artificial gaps at varying lengths: 1, 2, 4, 6, 8, 10, 12 and 18 hours. These gaps were added at five different intervals: 1 day, 1.5 days, 2 days, 3 days, and 5 days. One hundred simulations were run for each of these combinations. For each simulation, the rank was re-calculated for each modified vessel, within the original list, and the mean rank change for each simulation recorded for sensitivity to gaps.

### Case study

**Fig 1:**
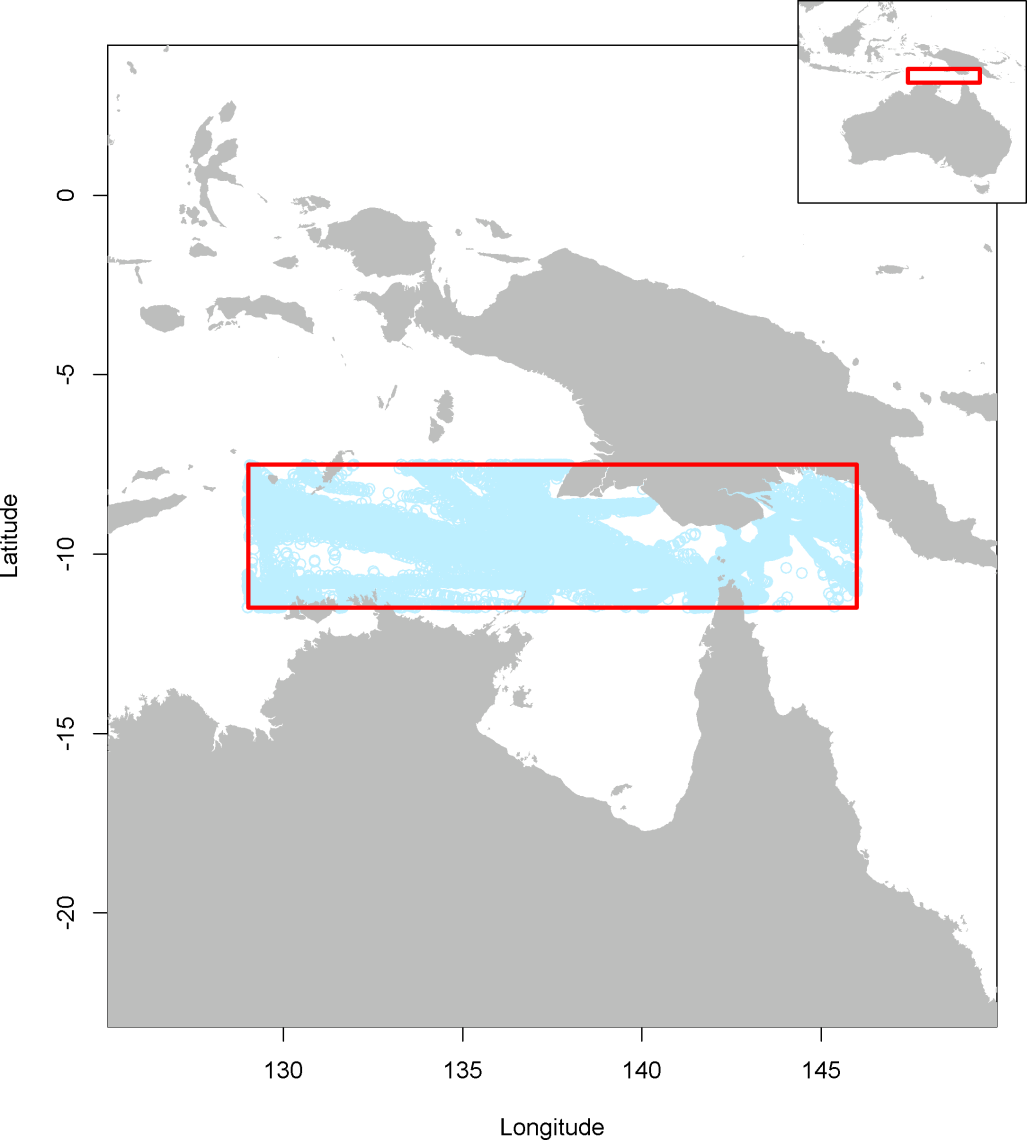
Geographic study area. The red box indicates the case study region used in this paper. Light blue dots indicate individual AIS transmissions for the month of September 2014.

The methods developed here are applied to a subset of AIS data from the case study area in the Arafura Sea where the Indonesian, Papua New Guinean and Australian Exclusive Economic Zone (EEZ) borders meet (Fig 1). There are several major ports in this region, and a major shipping lane joining Eastern Australia and Asia. This is an area known for high density of shipping, along with a high intensity of illicit activities including illegal fishing. Data were obtained from the Australian Maritime Safety Authority.

AIS data provides a wealth of information, but has many inherent data issues. Some of these issues include data overload to satellites, vessel operators intentionally switching off equipment, and errors in reported speed and time stamp. To build a model of expected polling rate, data was summarised and analysed as a daily summary per vessel (giving each vessel a count of polls per day). Prior to analysis, data was binned to hourly intervals–giving a maximum of 24 possible polls per 24 hour period. Data at one hour intervals was used to analyse an individual vessel’s transmission history to detect abnormal gaps. For each vessel, the average latitude and longitude for the day was used to indicate the approximate vessel location for that 24 hour period. To be conservative and to avoid modelling spurious data we removed gaps of more than five days.

There is a difference in reception rates between terrestrial and satellite receivers, with terrestrial receivers having reception rates an order of magnitude higher than satellites. Analysis of the 2014 data indicated that not only are there general differences in reception rates between receivers, there is also high temporal variance in reception rates for individual terrestrial receivers. Many terrestrial receivers show daily patterns and intermittent periods of inactivity. Therefore, we incorporated day into our model as a covariate to account for any temporal difference across the region.

## Results

### Model parameters and patterns

**Fig 2:**
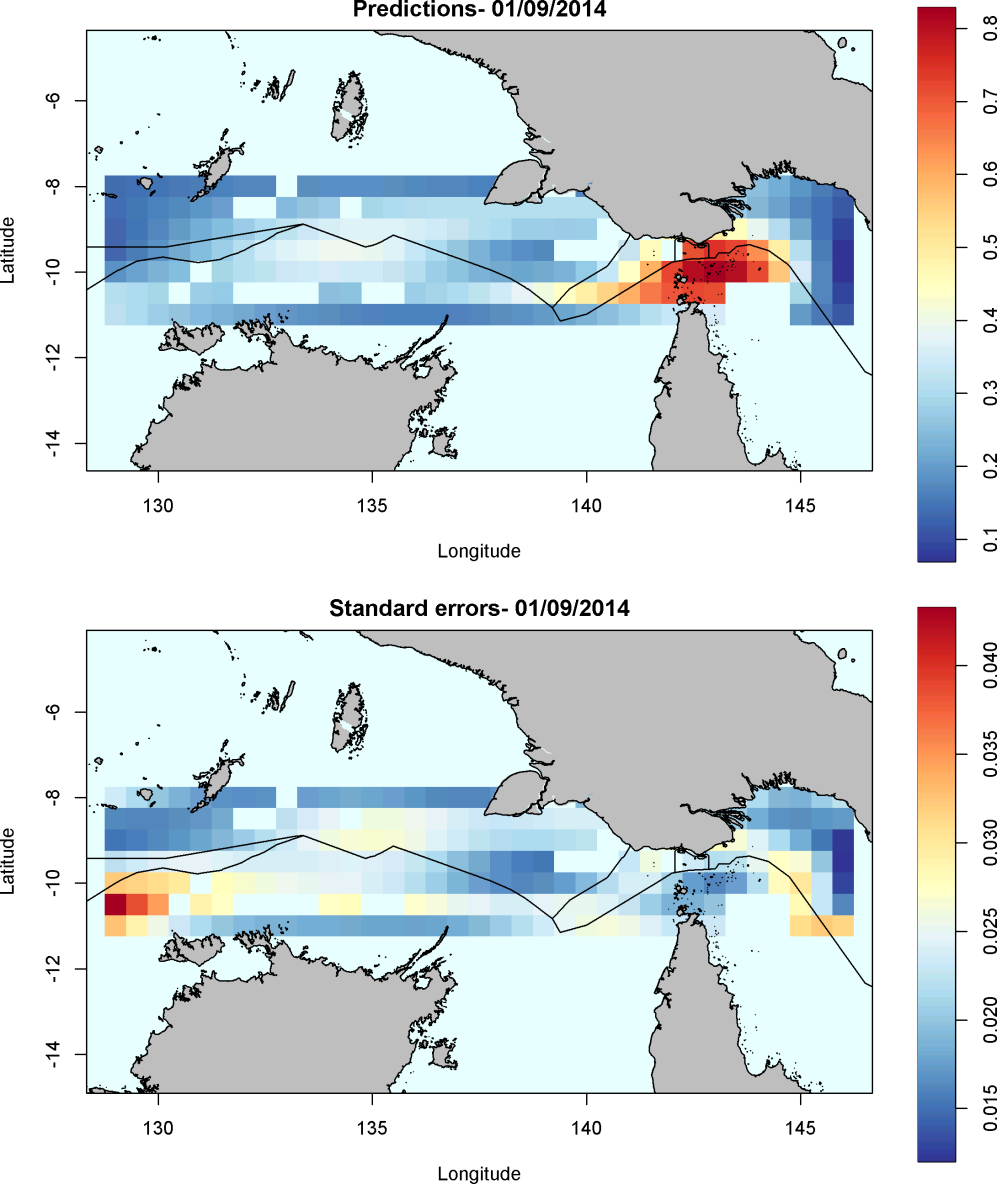
Model predictions and standard errors for AIS transmissions for the 1^st^ September 2014. **2A**) Model predictions, and (**2B**) standard errors, for the probability of a successful AIS transmission in a 60 minute window on 1^st^ September 2014. Note that high likelihood of frequent AIS transmission are seen in the dogleg, but low standard erros. In contrast, further West, the standard error for gap transmissions is high.

The GAM was fitted to 2000 vessel days across more than 400 vessels in the case study region for the month of September 2014. There is significant spatial variation in AIS transmission probabilities, which is compounded by changes in the spatial pattern over time (Fig 2A). Temporal and spatial changes to the probability of successful transmission surface are evident. The uncertainty within, and between days, is evident from the standard errors of the model surface (Fig 2B). Regions of low data, and more variable behavior, resulted in higher uncertainty in the probability of a transmission predicted by the model (Fig 2B).

Even within a small regional area such as this case study, regions of high and low probability are evident. Terrestrial stations are not present in the study region, however exploratory analyses in other regions indicate the model is able to detect changes around known terrestrial stations with variation evident both spatially and temporally.

### Vessel classification and robustness

The raw probabilities (PoG) give an indication of compliance and quality of a vessel, whereas the standardization (StdPoG) is designed to handle the situation in which a vessel has consistently unlikely gaps (for example if the AIS is set to transmit every hour). We found many vessels with AIS set to transmit at long intervals, for instance hourly. Based on the distribution of vessel transmission rates in our study area, high risk gaps were identified as those with a PoG of less than 5e-2, corresponding to a median length gap of around 36 hours.

Vessels with high risk gaps in the region included tug and pilot vessels (often with gaps near port), military vessels, and several vessels with known associations with Illegal Unreported and Unregulated (IUU) fishing activity. Multiple tug and pilot vessels were identified with shut offs in the region. This sensitivity indicates the model could be utilised for identification of vessels breaking compliance both at sea and in port. Given reliable vessel class type, this could be included as a covariate in the model.

Also of interest in this region is the areas along the EEZ boundaries–multiple high risk gaps were evident along the EEZ boundaries (e.g. Fig 2, at -10 ^o^, 138 ^o^). In particular, there appeared to be a hotspot for AIS shut-offs as vessels coming from the east crossed the Australian EEZ and entered the portion of the PNG EEZ projecting southwest and bordering the Indonesian EEZ.

**Fig 3:**
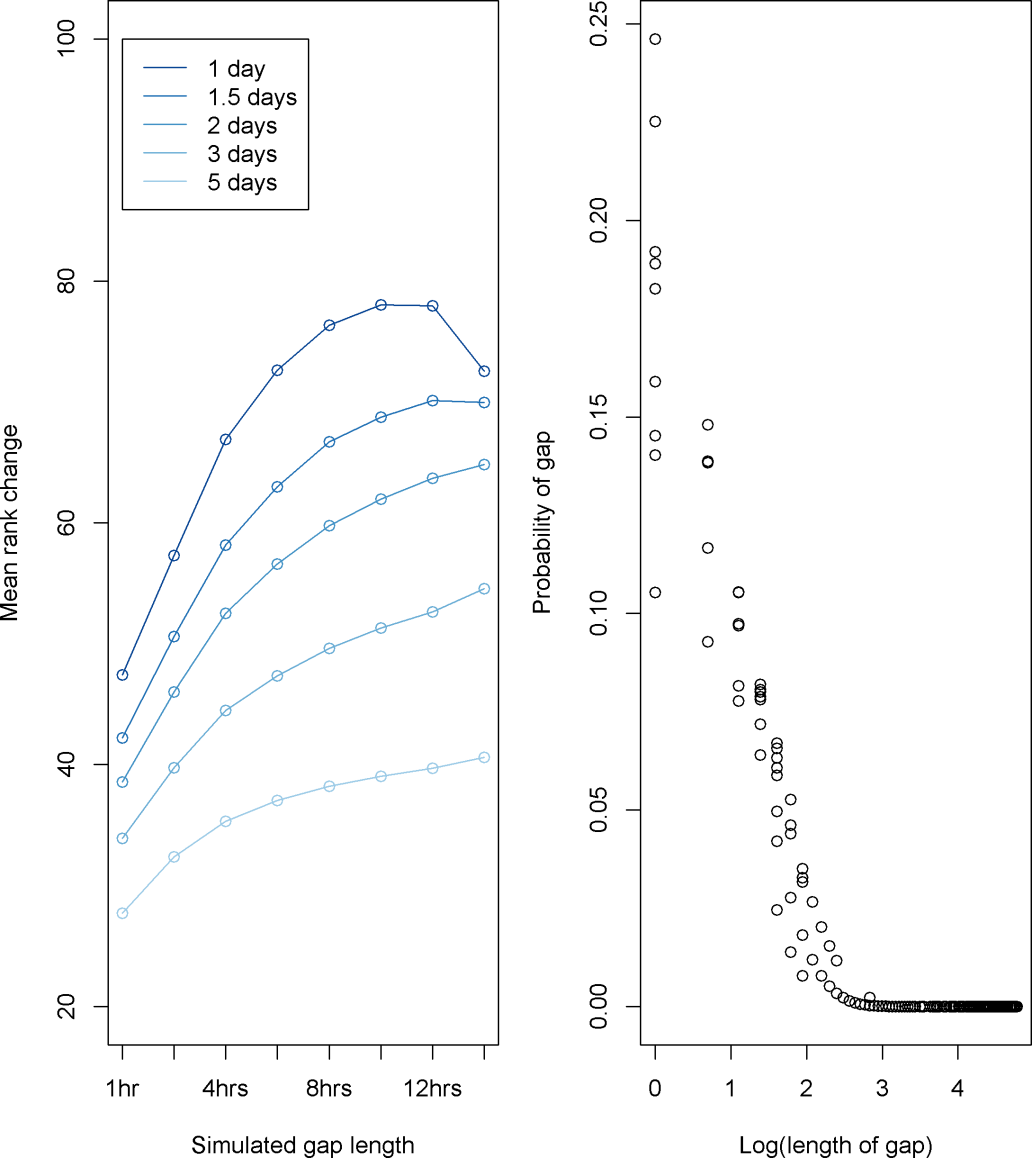
Simulation studies and gap probabilities. (**3A**) Simulation studies for addition of gaps of varying length at five different frequencies–circles show mean rank change for simulation. (**3B**) Plot of probability of a gap given (log) length of the gap shows expected decreasing probability of gap with increasing length.

Simulations indicate the PoG scores in the region were less sensitive to shorter gaps and most sensitive to gaps of 10–12 hours (Fig 3A). Sensitivity was gauged by rank change for vessels with inserted gaps–a positive change indicating an increase in a vessel’s rank for risk of anomalous gaps after addition of simulated gaps. For example, if vessel A was ranked 125th in the list of all vessels for risk of anomalous gaps–and then had a 6 hour gap inserted, every 24 hour period, it could potentially move up that same list to approximately position 50 (a positive rank change of 75). Across the simulations, mean rank change varied from 25 to 80. Decreasing probability of a gap given increase in length of gap was clearly evident in the results (Fig 3B).

The model can be used for identifying suspicious vessels in real time. Since notifications depend on the prediction step using a fitted model, these can be produced in real time or near real time.

### Case study application

**Fig 4:**
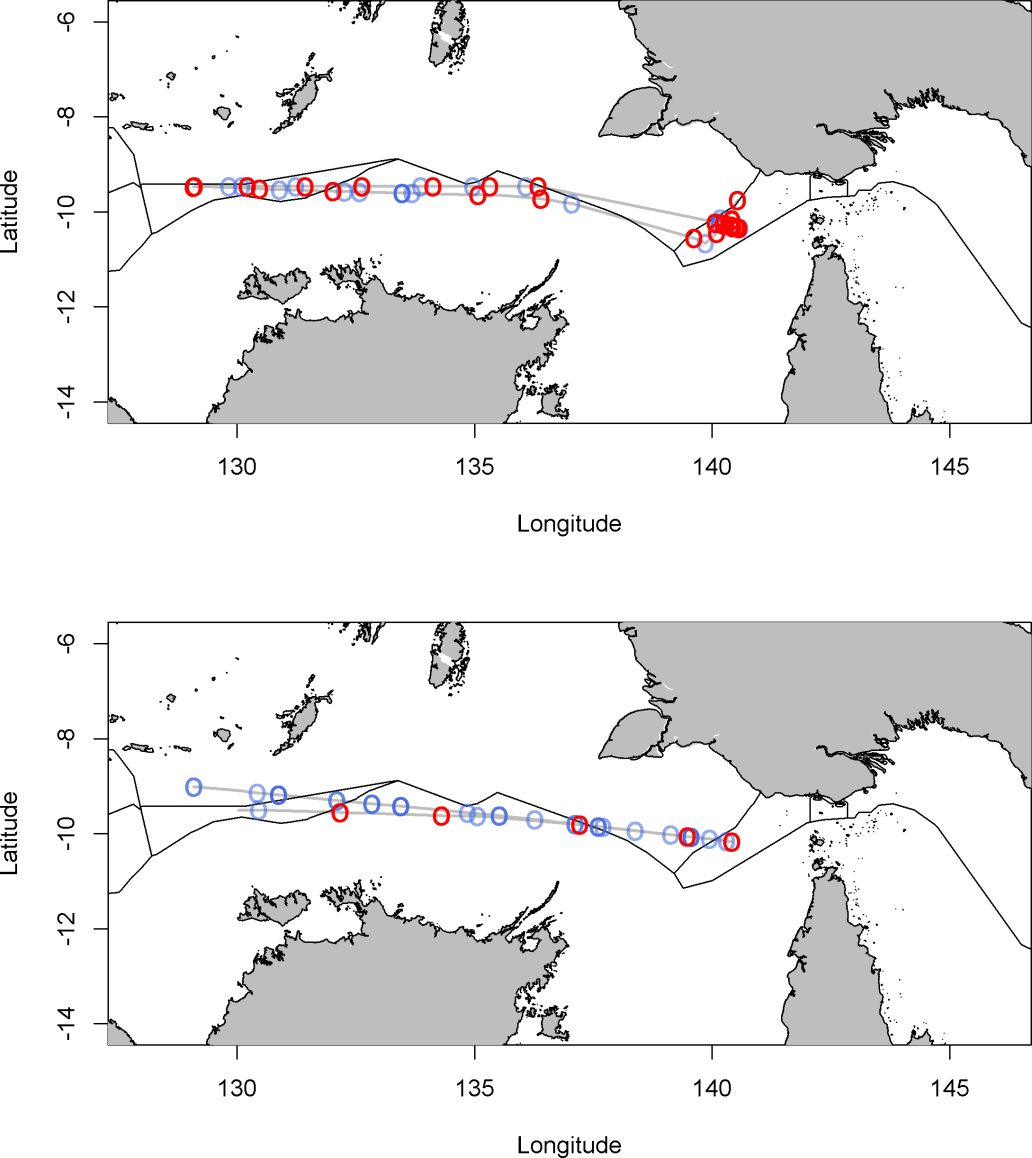
High and low risk gaps for two vessels. High risk gaps are shown in red, low risk gaps in blue. Each gap is indicated by an open circle ‘o’.

To give an indication of the derived results, we present results for two vessels identified with high-risk gaps. The first of these two vessels was traveling across the Australian and Indonesian EEZ border and registered multiple high-risk gaps, which are evident at locations close to the EEZ boundaries (Fig 4A). This vessel shows repeated patterns of high risk gaps starting and finishing on the western border of the tri-nation area known as the dogleg, where PNGs EEZ extends southwesterly in a linear extension (Fig 4A, at -10 ^o^, 140^o^). Frequent low risk gaps are also evident for this vessel, in general these low risk gaps are due to hourly AIS transmission. The high risk gaps however are due to unlikely longer gaps and located along the Australian and Indonesian EEZ. This particular vessel is a foreign flagged oil products tanker, and given the location and association with other vessels, is likely to be refueling IUU vessels. The second vessel, a foreign flagged reefer vessel, has multiple high risk gaps close to the Australian / Indonesian EEZ boundary, and again in the area known as the dogleg—an area known to have high rates of IUU (Fig 4B).

## Discussion

The development of a spatial model which enables effective identification of anomalous AIS transmission has a multitude of applications. To be effective, however, distinguishing the intentional disabling of AIS transmission from loss of transmission due to satellite saturation in high density areas or poor quality transmission is paramount. Our model was able to effectively distinguish abnormal transmission gaps, which enables us to rank vessels identified with high risk of potential intentional disabling of AIS transmissions. This is a critical first step for identifying vessels that warrant further investigation due to possible attempts to avoid observation.

There are a number of important applications that will benefit from the method we have developed. For instance, using the global AIS database we can assess whether vessels from a particular country of origin appear to be consistently disabling their AIS–either for long anomalous gaps, or regular but less frequent transmission. We can also identify geographic regions of high (or low) likelihood of disabling. These analyses provide enforcement opportunities, and can help with the allocation of resources to reduce illegal activities. This application is particularly relevant in light of the recently ratified United Nations FAO Port State Measures Agreement [14]. Under this agreement, port states commit to standardized inspection protocols for fishing vessels entering their ports. However, given the number of vessels operating and limited resources available for inspection, prioritizing high risk vessels will be critical. These analyses can also be applied to evaluate and compare the effectiveness of various government programs. For instance, in Indonesia, there have been numerous recent regulatory changes with regards to fishing behavior, the responses to which can be detected using the methods described here.

Using a spatial model such as a GAM has an advantage over simply empirically calculating average transmission rates in spatial grid cells as a basis for identifying anomalies. In contrast to other modelling approaches such as machine learning that require a training dataset of known patterns, a labeled training dataset is not required to use our model. A second advantage is that the model explicitly considers correlation among neighbouring cells, unlike other approaches that treat each location as independent (e.g. [15]). Since by their nature GAMs allow spatial dependency, if there is little or no data at a certain location, strength is gained by incorporating information from nearby locations. This allows the model, within reason, to predict expected transmission rates in areas where little data has been collected, smoothing out oddities where one vessel is operating in a cell. This is an important distinction of our approach, as an empirical distribution for the cell would deem the behaviour normal by definition, as the vessel’s transmission rate in the cell would be the only observation from which to form the empirical estimate. In the approach developed here, the biggest limitation we forsee is the normalisation of abnormal behaviour. For example, if in a given area, all vessels turn off their AIS, this might be seen by the model as normal behaviour for vessels in that area. However, this problem is avoided to some extent by the continuous nature of the smoothing in the model.

Our simulation evaluation of the model showed that the model is responsive to vessels with gaps on the order of a day (24 hour period). Moreover, it is less sensitive to shorter gaps. This is an encouraging finding, as many vessels have their AIS set to transmit at hourly intervals and our data was binned to one hour intervals, and identifying gaps of this length or shorter as anomalous would indicate the model is not adequately selective. After addition of varying length gaps, the simulations showed mean positive rank change—indicating increased risk for anomalous gaps.

Illegal activities on the water are notoriously hard to observe, particularly if there is a risk of enforcement action. Risk-based approaches such as the one we have developed here will be critical to inform enforcement opportunities; they can provide indications of likely illicit behaviour that can be followed up with either further investigation or enforcement action. We developed a model that can be widely applied, is fast and easy to use, and can give probabilistic predictions that can be easily used in a risk context.

This model also allows for vessel-specific behaviour to be identified and further investigation followed. For instance, our model can readily be used to ask whether a vessel’s behavior changes over time, across locations, or after interventions. Our model frame can also include other information on vessels or locations, such as vessel class, size, ownership, or proximity to features like international boundaries or high value fisheries. Even as vessels respond to the increasing use of AIS as a monitoring tool, historical analysis of vessel transmissions using the model we present can be informative for evaluating future behavior and risk.

As international efforts to address illegal fishing intensify, including Regional Plans of Action along with the newly ratified Port State Measures Agreement, it will be important that countries are able to evaluate the activities of vessels entering or operating in their waters. Given the number of industrial scale vessels globally, discovering and prioritizing high risk vessels will be a key need. This statistical tool can provide a backbone for this type of service and it can be used to process the global AIS feed in real time, allowing national coast guards and fisheries agencies, along with international organizations, to scan the vast volume of AIS data and prioritize vessels for further investigation or enforcement action in real time. Inclusion of additional covariates such as ship type is possible, however such data derived from AIS messages are often unreliable. This tool could be combined with additional risk based models, for example of anomalous vessel movement and proximity to fishing locations, to provide a comprehensive risk framework. This is critically important given that IUU is one of the main fisheries issues threatening both stock sustainability and broader environmental impacts. Increasing our understanding of IUU is a key activity for ensuring economically productive and environmentally friendly fisheries [16].
